# The Modern Treatment of Typhoid

**Published:** 1897-02

**Authors:** 


### THE MODERN TREATMENT OF TYPHOID.

The fact that the most incurable ailments boast of the largest assortment of remedies is strikingly illustrated in the case of this fever.

Each year a remedy is proposed and tested until the next appears. Many of these are harmful—some are absolutely useless, and a very few are of some value.

The Brandt method belongs to the latter class, for by its action on the heat centers and the temperature, it is capable of reducing temperature and influencing favorably the mortality of the disease.

However, it has many disadvantages in being in itself objectionable to many. Its use entails considerable expense and careful, intelligent supervision on the part of the physician and attendant.

Hence its use is decidedly limited. Moreover, there is no evidence to show that in mild cases of the disease it possesses any advantages over the older methods of treatment.

The Woodbridge, or the various antiseptic methods of recent date, can not be said to possess any decided remedial qualities. On the other hand, we have often thought that antiseptics were capable of doing harm by their effects upon digestion and assimilation.

It is reasonable to infer that auto-infection can be prevented far better by proper choice of nutritives and by free elimination than by the employment of any agent which is designed to act in a purely local manner.

By the way, what chemical antidote have we for a ptomaine or leucomaine in the lower half of the intestinal tract?

So we again return to a rational symptomatic treatment of the disease, having found that no one agent is invariably effective, but that all of them may at times have a place in the management of the affection.—*N. E. Medical Monthly.*

---

### STATISTICS OF MEDICAL MEN.

It is said that Russia with its population of 110,000,000 has only 18,334 physicians, or one to each 6000 inhabitants! whereas in Germany, France, and England the ratio is one to 3000, 1800, and 1500, respectively. In other words, there are twice as many medical men and women in proportion to the total population in Germany as there are in Russia; and four times as many in England as in Russia, and twice as many in Germany, France having more than Germany and fewer than England.

There are in the United States, with its population of 75,000,000, 120,000 physicians.

The European land in which the proportion of physicians and surgeons is greatest is Scotland. The European country in which, according to the population, physicians are least numerous is Russia, and next to it in the list is Italy. There are, in proportion to the population, more doctors in Ireland than in England, and many more in Spain than in Italy.

